# Combinations of Quality and Frequency of Immunization Activities to Stop and Prevent Poliovirus Transmission in the High-Risk Area of Northwest Nigeria

**DOI:** 10.1371/journal.pone.0130123

**Published:** 2015-06-11

**Authors:** Radboud J. Duintjer Tebbens, Mark A. Pallansch, Steven G. F. Wassilak, Stephen L. Cochi, Kimberly M. Thompson

**Affiliations:** 1 Kid Risk, Inc., Orlando, Florida, United States of America; 2 Division of Viral Diseases, National Center for Immunization and Respiratory Diseases, Centers for Disease Control and Prevention, Atlanta, Georgia, United States of America; 3 Global Immunization Division, Center for Global Health, Centers for Disease Control and Prevention, Atlanta, Georgia, United States of America; National Institute for Public Health and the Environment, NETHERLANDS

## Abstract

**Background:**

Frequent supplemental immunization activities (SIAs) with the oral poliovirus vaccine (OPV) represent the primary strategy to interrupt poliovirus transmission in the last endemic areas.

**Materials and Methods:**

Using a differential-equation based poliovirus transmission model tailored to high-risk areas in Nigeria, we perform one-way and multi-way sensitivity analyses to demonstrate the impact of different assumptions about routine immunization (RI) and the frequency and quality of SIAs on population immunity to transmission and persistence or emergence of circulating vaccine-derived polioviruses (cVDPVs) after OPV cessation.

**Results:**

More trivalent OPV use remains critical to avoid serotype 2 cVDPVs. RI schedules with or without inactivated polio vaccine (IPV) could significantly improve population immunity if coverage increases well above current levels in under-vaccinated subpopulations. Similarly, the impact of SIAs on overall population immunity and cVDPV risks depends on their ability to reach under-vaccinated groups (i.e., SIA quality). Lower SIA coverage in the under-vaccinated subpopulation results in a higher frequency of SIAs needed to maintain high enough population immunity to avoid cVDPVs after OPV cessation.

**Conclusions:**

National immunization program managers in northwest Nigeria should recognize the benefits of increasing RI and SIA quality. Sufficiently improving RI coverage and improving SIA quality will reduce the frequency of SIAs required to stop and prevent future poliovirus transmission. Better information about the incremental costs to identify and reach under-vaccinated children would help determine the optimal balance between spending to increase SIA and RI quality and spending to increase SIA frequency.

## Introduction

Countries use a wide variety of immunization strategies for polio.[1] Routine immunization (RI) programs vaccinate children according to an age-specific schedule throughout the year, while supplemental immunization activities (SIAs, e.g., national immunization days and outbreak response campaigns) typically target a wide age range of children (e.g., 0–4 year olds) regardless of vaccination status during a limited period of time, often linked to epidemiologic assessment of risk for poliovirus transmission.[2, 3] The Global Polio Eradication Initiative (GPEI) relies heavily on SIAs with oral poliovirus vaccine (OPV) to rapidly increase population immunity, interrupt chains of transmission, and immunize children missed by RI.[4] Countries with GPEI partner support eliminated naturally occurring wild poliovirus (WPV) serotype 2 (WPV2) by 2000,[5] achieved apparent global interruption of WPV serotype 3 (WPV3) transmission in 2012,[6] and limited indigenous transmission of WPV serotype 1 (WPV1) to decreasing areas in three countries (Afghanistan, Pakistan, Nigeria).[7] However, challenges remain to interrupt the last chains of WPV transmission in difficult-to-reach areas in these countries despite very frequent SIAs, and many outbreaks due to exported WPVs occurred in previously polio-free countries in the last decade.[8] Relatively lower seroconversion of OPV in some areas [1, 9] contributes to the need for more SIAs. However, SIAs may also repeatedly vaccinate the same accessible children while missing unvaccinated children in some communities. In addition to vaccine coverage, population immunity to transmission depends on the mixing and numbers of under-vaccinated individuals.[10, 11]

National immunization and GPEI leaders continue to seek the most cost-effective uses of their substantial, but finite, immunization program resources. Achieving WPV elimination in a country requires increasing population immunity high enough to interrupt transmission. However, once individual countries eliminate WPVs, they remain at risk of importing WPVs from elsewhere until global eradication occurs,[12] and consequently achieving eradication requires that WPV-free countries maintain high population immunity until all countries have interrupted WPV transmission. Using OPV and failing to maintain high population immunity leads to some additional potential consequences. Specifically, populations with low immunity levels can sustain the transmission of the live, attenuated OPV virus beyond immediate contacts of vaccine recipients and allow it to establish sustained transmission to ultimately result in outbreaks of paralysis from circulating vaccine-derived poliovirus (cVDPV).[13–15] The reality of cVDPVs as well as the very low but noticeable rate of vaccine-associated paralytic polio (VAPP) in OPV recipients and close contacts motivate plans to discontinue all OPV use after WPV eradication.[16, 17] The GPEI currently plans to coordinate the cessation of all serotype-2-containing OPV use (OPV2 cessation) in April 2016, followed by cessation of all remaining OPV serotypes (OPV13 cessation) as soon as 2019 if WPV transmission stops at least three years before that time.[4] Currently, trivalent OPV (tOPV) remains the only serotype-2-containing OPV in use, with serotype 2 monovalent OPV (mOPV2) representing a possible vaccine for outbreak response in the event of a serotype 2 outbreak after OPV2 cessation. Serotype 1- and 3- containing OPV include mOPV1 and mOPV3, respectively, as well as tOPV and bivalent serotype 1 and 3 OPV (bOPV).

Northwest Nigeria historically represents one of the key poliovirus reservoirs, with large outbreaks of WPV1, cVDPV2, and WPV3 reported during the past decade and exported poliovirus leading to outbreaks in previously polio-free countries on numerous occasions.[12, 18, 19] Increased quality and frequency of SIAs in northwest Nigeria in recent years contributed to probable elimination of WPV3 and only 5 reported WPV1-confirmed polio cases in 2014.[7] However, most SIAs since 2010 used bOPV. While trivalent OPV (tOPV) remains in use for RI, coverage with 3 or more tOPV doses in northwest Nigeria remains extremely low according to the most recent survey.[20] The limited use of tOPV for SIAs allows accumulation of susceptible children in northwest Nigeria and continued poliovirus transmission and cases associated with a cVDPV2 outbreak that started in 2005.[21, 22] Using a model for poliovirus transmission northwest Nigeria, we previously showed the importance of better reaching the under-vaccinated communities while maintaining high vaccination intensity in the general population to achieve and maintain sufficient population immunity to transmission for all 3 serotypes.[10, 23] Health authorities currently plan more frequent tOPV SIAs in northwest Nigeria leading up to coordinated OPV2 cessation (e.g., 4 tOPV SIAs during 2015), but because population immunity drops once homotypic OPV use stops, preventing subsequent cVDPV emergences requires higher population immunity at the time of OPV cessation than the threshold needed to stop WPV and cVDPV transmission.[24] Thus, both quality and frequency of SIAs with different vaccines influence serotype-specific population immunity to transmission, the ability to achieve and maintain WPV-free status, and the ability to successfully manage OPV2 cessation. This study explores how different assumptions about the quality of RI and the quality and frequency of SIAs affect population immunity and the possibility of cVDPV outbreaks after OPV cessation. We identify the model assumptions related to immunization quality that most affect population immunity of all three serotypes. Given the current plans for OPV2 cessation during 2016, we then focus the analysis of combinations of quality and frequency only on tOPV SIAs.

## Material and Methods

We previously developed a dynamic differential-equation based model of poliovirus transmission and OPV evolution of northwest Nigeria [25] and used it to address vaccination policy questions.[10, 23, 26] We use the same model and adopt all model inputs and assumptions from the most recent model update that incorporates new data on RI coverage and SIA plans.[26] We define the reference case as the model run that assumes constant model input assumptions and expected SIA frequency going forward. The model input values reflect the result of an extensive expert elicitation and model calibration process across multiple settings to reproduce behavior consistent with the evidence, including paralytic incidence, WPV die-out, cVDPV emergence and outbreaks (or absence thereof in some settings), vaccination coverage surveys and data on missed children, and age distributions of cases.[15, 23, 25, 27, 28] The reference case further assumes OPV2 cessation on April 1, 2016, consistent with the current GPEI plan,[4] although we previously reported that the expected time until cVDPV2 elimination in northwest Nigeria and elsewhere may necessitate delaying OPV2 cessation.[26] The reference case assumes OPV13 cessation occurs on April 1, 2019. The model divides the approximately 45 million people in the northwest zone of Nigeria comprising 7 states [25, 29] into the general population (90%) and an under-vaccinated subpopulation (10%) characterized by relatively lower RI and SIA coverage and preferential subpopulation mixing. The model further accounts for seasonality in transmission, which explain the historically highest incidence during the summer months,[30] by varying R_0_ throughout the year.[23, 25]

We performed an exploratory sensitivity analysis to characterize the impacts of changing RI and SIA model inputs on population immunity to transmission and cVDPV risks. We varied individual inputs starting on January 1, 2015 while keeping all others at reference case values, although in instances with potentially important interactions we varied two inputs simultaneously (e.g., RI coverage in both the general population and the under-vaccinated subpopulation). We explored the baseline RI coverage, which represents the coverage with 3 or more non-birth RI doses of tOPV (until OPV2 cessation in April 2016) or bOPV (after OPV2 cessation) in the general population (i.e. POL3). As we vary the baseline RI coverage, which equals approximately 0.14 in the reference case (based on the most recent RI coverage survey in Nigeria [20]), we proportionately vary the birth dose coverage and the partial coverage with 1 or 2 non-birth doses, so that the baseline RI coverage linearly affects the overall effective vaccination coverage due to RI.[25] Using baseline RI coverage as a measure of overall RI immunization performance, RI coverage in the under-vaccinated subpopulation depends directly on the baseline RI coverage through multiplication by the relative RI coverage in the subpopulation, which equals 0.3 in the reference case (based on prior modeling of this population [10, 23, 25, 26]). The reference case assumes no IPV use, but the exploratory analysis considers the policy of administering an IPV dose simultaneously with the 3^rd^ scheduled non-birth RI OPV dose. We assume that children who receive at least one non-birth OPV dose by 3 months of age would also receive IPV by that time as described elsewhere.[10, 31] The 3 model inputs related to SIAs that we varied include the baseline true SIA coverage in the general population (0.85 in the reference case), the baseline repeated missed probability in the general population (0.85 in the reference case), and the relative SIA coverage (RSC) in the under-vaccinated subpopulation compared to the general population (0.2 in the reference case).[23] The true SIA coverage represents the overall fraction of children under 5 years of age who receive a dose during a single SIA, while the repeated missed probability characterizes the fraction of children targeted but missed by the previous SIA that will not receive a dose in the current SIA.[23] Similar to RI immunization, the baseline values of the general population represent overall SIA performance and proportionally affect the SIA impact in the under-vaccinated subpopulation through the RSC.[23]

We focus on two key outcomes for each serotype: 1) population immunity to transmission on January 1, 2016 (i.e., after applying a change in vaccination-related model inputs for one year and shortly before planned OPV2 cessation), and 2) circulation of cVDPVs beyond one year after serotype-specific OPV cessation, defined as prevalence of fully-reverted polioviruses at least one year after homotypic OPV cessation above the minimum level for which the model assumes any poliovirus can generate a non-zero force-of-infection.[25] We characterize population immunity using the mixing-adjusted effective immune proportion (EIPM = 1-R_n_/R_0_, where R_0_ is the basic reproductive number).[10] However, EIPM varies seasonally and by serotype because it depends on R_0_, and therefore we used R_n_ as a universal, scaled measure of population immunity for our analyses. We defined the mixing-adjusted net reproductive number (R_n_) as the average number of secondary infections generated by a single infection taking into account heterogeneous mixing between age groups and subpopulations and contributions from individuals in all immunity states.[10] Above the threshold (i.e., denoted R_n_* and equal to 1) each new infection will generate more new infections and an outbreak can occur, while if R_n_ remains below R_n_* for long enough, then transmission will die out. Circulation of cVDPVs one year or more after homotypic OPV cessation may result from failure to interrupt cVDPV transmission before OPV cessation, or the creation of a new cVDPV emergence after OPV cessation due to insufficient population immunity at the time of homotypic OPV cessation to prevent the occurrence of a cVDPV outbreak.[24] The reference case for the model fails to interrupt the transmission of cVDPVs prior to OPV2 cessation.

The results of the exploratory sensitivity analysis led us to focus on the RSC as a primary metric for SIA performance. We performed additional analyses with fixed true coverage and repeated missed probability for each SIA round of 85% for the general population and we emphasize that maintaining this high coverage remains critical in the context of managing OPV cessation of each serotype. We simultaneously varied RSC and the number of tOPV rounds, while keeping the total annual number of tOPV and bOPV SIAs fixed at 9, and reported the impacts on the R_n_ for serotype 2 on January 1, 2016 and whether cVDPV2s circulate for a year or more after OPV2 cessation. For any given annual number of tOPV SIAs between 1 and 6, we used 1% increments in RSC to determine the minimum RSC required to prevent cVDPV2s after OPV2 cessation. We repeated the analysis for different assumptions about RI coverage from 2015 and beyond.

## Results

**Fig 1:**
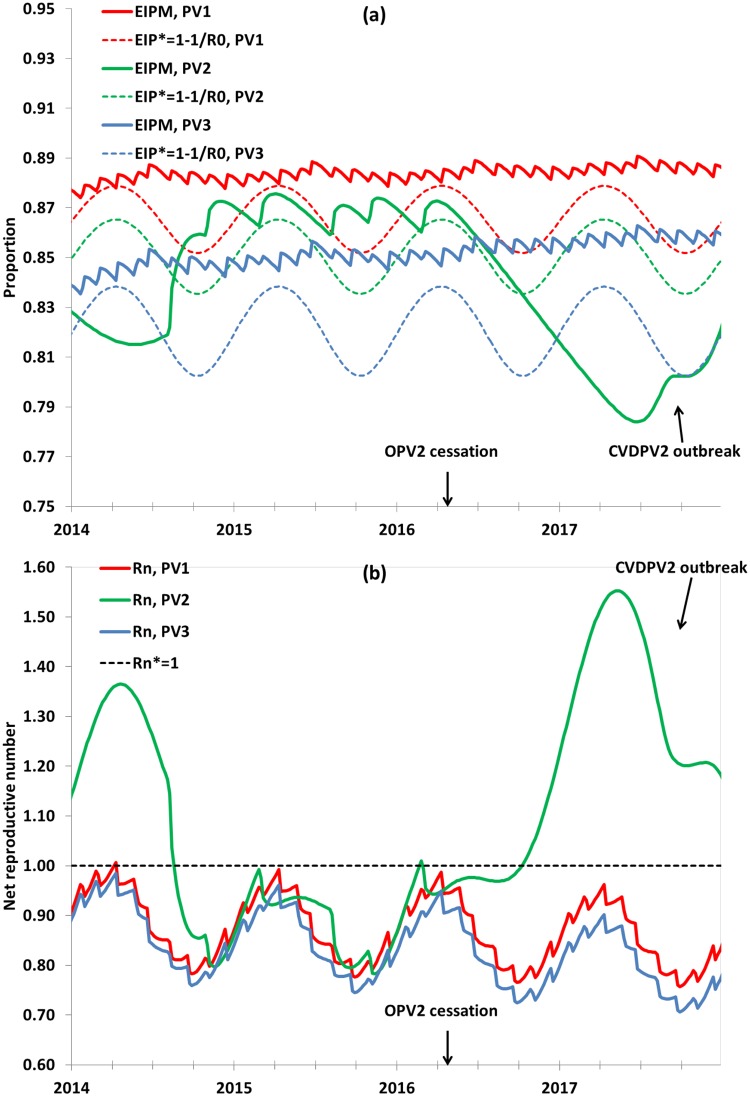
Population immunity in northwest Nigeria for the reference case, 2014–2017. (a) Mixing-adjusted effective immune proportion (EIPM) compared to the threshold (EIP*). (b) Mixing-adjusted net reproductive number (R_n_) compared to threshold of R_n_* = 1.

Fig 1 shows the population immunity in northwest Nigeria between 2014 and 2018 for the reference case, both in terms of EIPM and R_n_. Serotype 3 population immunity exceeded the threshold from the start, serotype 1 population immunity reached and sustained high enough levels in 2014, and serotype 2 population immunity became insufficient in 2014 following a long period of time without any tOPV SIAs, consistent with the increase in cVDPV2 cases reported that year.[32] With the projected 6 total tOPV SIAs in 2014 and 2015, respectively, and immunity associated with the cVDPV2 outbreak, population immunity reaches the threshold, but remains close enough to sustain a low level of silent cVDPV2 circulation until after OPV2 cessation. In the absence of OPV2 vaccine after OPV2 cessation, population immunity drops further until eventually a large cVDPV2 outbreak occurs, which would necessitate an outbreak response with mOPV2 or tOPV from the global stockpile. With insufficient population immunity to transmission at the time of OPV2 cessation, subsequent cVDPV2s could occur both due to continued silent circulation of current cVDPVs or due to the development of new cVDPV2s derived from tOPV used just prior to OPV2 cessation.[24] Programmatically, both of these events would represent a public health emergency, but more tOPV use to increase population immunity to transmission at the time of OPV2 cessation can prevent them.[24]

Table 1 shows the impact of varying model inputs related to RI starting January 1, 2015. Changes around the currently very low RI coverage in the reference case lead to only small impact on population immunity. However, given that the reference case attains serotype 2 population immunity slightly below the level needed to avoid re-emergence of cVDPV2s after OPV2 cessation, small increases in population immunity can prevent cVDPV2s after OPV2 cessation. For example, if the baseline RI coverage in the general population increases to 0.5, which also increases the RI coverage in the subpopulation to 0.5×0.3 = 0.15, then cVDPV2s do not persist after OPV2 cessation despite the tail end of the ongoing cVDPV2 outbreak lasting up until OPV2 cessation. Seasonality plays a role in interrupting cVDPV2 transmission despite no OPV2 vaccine, because the average number of secondary transmissions per new infection becomes lower in the low season (i.e., as a result of the lower R_0_ during the low season) (Fig 1). If both the baseline RI coverage and the relative RI coverage in the under-vaccinated subpopulation increase to 0.5, then this implies a significant improvement in absolute RI coverage for the under-vaccinated subpopulation to 0.25. This improvement results in a marked reduction in R_n_, interrupts cVDPV2 transmission by January 1, 2016, and avoids cVDPV2s after OPV2 cessation. Consistent with prior work,[10, 33] the addition of a single IPV RI dose given with the third dose of OPV in RI from January 1, 2015 does not yield a large impact on population immunity or on cVDPV circulation after OPV cessation due to the low RI coverage in northwest Nigeria.[25]

**Table 1:** Exploratory sensitivity analysis of model inputs and assumptions that characterize routine immunization (RI) (reference case shown in italics).

Varied input(s), value(s)	R_n_ at 1/1/2016			cVDPVs ater OPV cessation?^a^		
	Type 1	Type 2^b^	Type 3	Type 1	Type 2	Type 3
**Baseline RI coverage (general population):**						
0.1	0.87	0.87*	0.83	No	**Yes**	No
*0*.*14 (reference)*	*0*.*87*	*0*.87*	*0*.*83*	*No*	***Yes***	*No*
0.3	0.83	0.82*	0.81	No	No	No
0.5	0.80	0.77*	0.78	No	No	No
**Relative RI coverage in the under-vaccinated subpopulation:**						
0	0.89	0.91*	0.85	No	**Yes**	No
0.1	0.88	0.89*	0.84	No	**Yes**	No
*0*.*3 (reference)*	*0*.*87*	*0*.87*	*0*.*83*	*No*	***Yes***	*No*
0.5	0.85	0.84*	0.82	No	No	No
**Baseline and relative RI coverage combined:**						
*Baseline RI coverage = 0*.*139*, *relative RI coverage = 0*.*3(reference)*	*0*.*87*	*0*.87*	*0*.*83*	*No*	***Yes***	*No*
Baseline RI coverage = 0.5, relative RI coverage = 0.5	0.75	0.70	0.75	No	No	No
**IPV use for RI:**						
*No IPV doses added to RI schedule (reference)*	*0*.*87*	*0*.87*	*0*.*83*	*No*	***Yes***	*No*
1 IPV dose added to RI schedule from 1/1/2015	0.86	0.87*	0.83	No	**Yes**	No

^a^ Defined as occurrence of fully-reverted virus prevalence above the transmission threshold (EPI*), including due to cVDPV emergence after OPV cessation, or failure to interrupt cVDPV transmission before OPV cessation.

^b^ Asterisk indicates continued cVDPV2 transmission at 1/1/2016, but at such low levels that it has negligible impact on R_n_ at January 1, 2016.

Table 2 shows how SIA-related model inputs influence population immunity and cVDPV risks after OPV cessation. The baseline true SIA coverage in the general population, which directly influences the under-vaccinated subpopulation through the RSC, only moderately influences population immunity. However, a very high baseline repeated missed probability implies more susceptible children both in the general and under-vaccinated subpopulations resulting in markedly lower population immunity. We see that the largest impact on population immunity comes from increasing coverage in the under-vaccinated subpopulation (i.e., increasing the RSC), with R_n_ ranging from 0.63 for PV3 with RSC = 0.5 (i.e., very high population immunity) to approximately 1 or more for all 3 serotypes with RSC = 0.1. An increase of RSC from 0.2 in the reference case to 0.25 prevents the cVDPV2s after OPV2 cessation.

**Table 2:** Exploratory sensitivity analysis of model inputs and assumptions that characterize SIAs (reference case shown in italics).

Varied input(s), value(s)	R_n_ at 1/1/2016			cVDPVs afterOPV cessation?^a^		
	Type 1	Type 2^b^	Type 3	Type 1	Type 2	Type 3
**Baseline true SIA coverage (general population):**						
0.75	0.90	0.89*	0.85	No	**Yes**	No
0.80	0.88	0.88*	0.84	No	**Yes**	No
*0*.*85 (reference)*	*0*.*87*	*0*.87*	*0*.*83*	*No*	***Yes***	*No*
0.90	0.85	0.86*	0.82	No	**Yes**	No
0.95	0.84	0.85*	0.80	No	No	No
**Baseline repeated missed probability (general population):**						
0.75	0.84	0.85*	0.80	No	*No*	No
*0*.*85 (reference)*	*0*.*87*	*0*.87*	*0*.*83*	*No*	***Yes***	*No*
0.95	0.95	0.90*	0.90	No	**Yes**	No
**Relative SIA coverage in the under-vaccinated subpopulation (RSC):**						
0.1	1.02	0.96*	0.97	No	**Yes**	No
0.15	0.93	0.91*	0.89	No	**Yes**	No
*0*.*2(reference)*	*0*.*87*	*0*.87*	*0*.*83*	*No*	***Yes***	*No*
0.25	0.81	0.83*	0.78	No	No	No
0.30	0.77	0.80*	0.74	No	No	No
0.50	0.65	0.70	0.63	No	No	No

^a^ Defined as occurrence of fully-reverted virus prevalence above the transmission threshold (EPI*), including due to cVDPV emergence after OPV cessation, or failure to interrupt cVDPV transmission before OPV cessation.

^b^ Asterisk indicates continued cVDPV2 transmission at 1/1/2016, but at such low levels that it has negligible impact on R_n_ at 1/1/2016.

None of the changes in RI and SIA model inputs we considered resulted in cVDPV1 or cVDPV3 outbreaks after OPV13 cessation, despite similar population immunity levels to transmission on January 1, 2016, because serotype 1 and 3 population immunity to transmission continues to increase after OPV2 cessation with all RI and SIA bOPV use, and because our model assumes that serotype 1 and 3 OPV revert more slowly and remain relatively less transmissible than serotype 2 OPV.[15, 23, 25, 27, 28]

**Fig 2:**
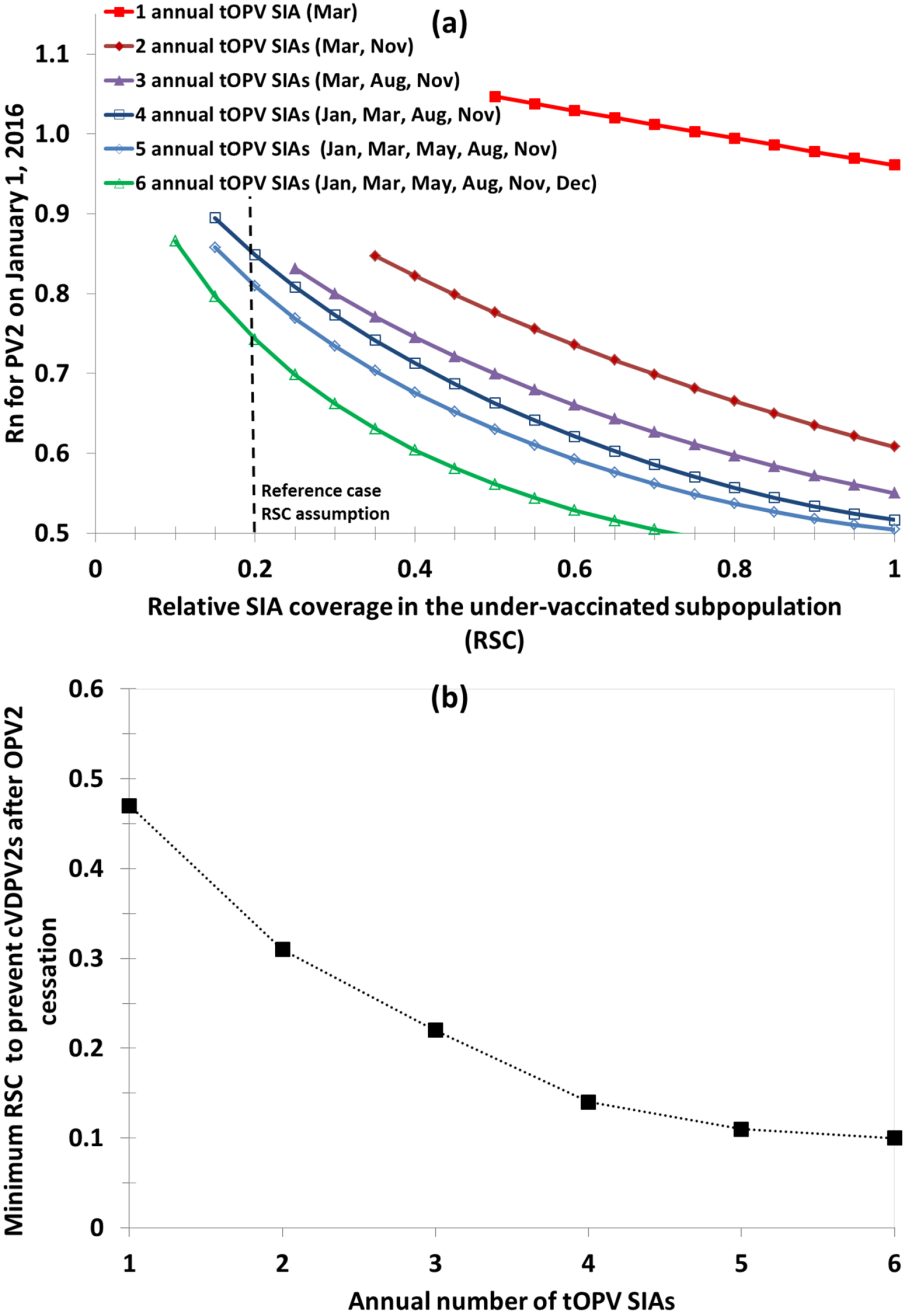
Interactions between tOPV SIA quality and frequency leading up to OPV2 cessation in northwest Nigeria. Acronyms: cVDPV, circulating vaccine-derived poliovirus; OPV2, serotype 2-containing oral poliovirus vaccine; R_n_, net reproduction number; RSC, relative SIA coverage in the under-vaccinated subpopulation; SIA, supplemental immunization activity; tOPV, trivalent oral poliovirus vaccine. (a) Impact on R_n_ for type 2, omitting points for which cVDPV2s circulate beyond a year after OPV2 cessation. (b) Minimum RSC to prevent cVDPVs after OPV2 cessation as a function the annual number of tOPV SIAs.

Fig 2 illustrates the interactions between RSC and the annual number of tOPV rounds in terms of population immunity and cVDPV2 risks after OPV2 cessation. The curves in Fig 2a omit higher values of R_n_ for which population immunity remains insufficient to prevent persistent cVDPV2s after OPV2 cessation, because these situations represent programmatic failures. Increasing the number of tOPV SIAs clearly increases population immunity to serotype 2 (i.e., reduces R_n_), with a larger increase between 1 and 2 SIAs than between 2 and 3 SIAs. The distance between the curves also depends on the timing of the SIAs. For example, the distance between the curves for 4 and 5 tOPV SIAs remains very small because for both scenarios the last two SIAs conducted prior to January 1, 2016 remain the same (i.e., August and November 2015). Similarly, the curve for 6 tOPV SIAs includes a December SIA, just prior to the time when we record R_n_ on January 1, 2016. For low values of RSC, population immunity can still remain very low despite high numbers of tOPV SIAs. For the reference case assumption of RSC = 0.2, 3 tOPV SIAs do not prevent cVDPVs after OPV2 cessation. This suggests the need for at least 5 tOPV SIAs from early 2015 through OPV2 cessation to prevent cVDPV2s if RSC does not improve (i.e., 4 in 2015 and 1 in March 2016). Fig 2b shows the minimum RSC needed to prevent persistent cVDPV2s after OPV2 cessation as a function of the number of tOPV SIAs. For example, only 1 annual tOPV SIA with an RSC of at least 0.5 from 2015 forward (including in March 2016, shortly before OPV2 cessation) appears sufficient to prevent cVDPV2 persistence. However, with 4 tOPV SIAs, preventing cVDPV2s after OPV2 cessation requires an RSC of only 0.15 or more. The nonlinear relationship underscores the importance of reaching the under-vaccinated subpopulation, with increasingly frequent tOPV SIAs needed as RSC decreases.

**Fig 3:**
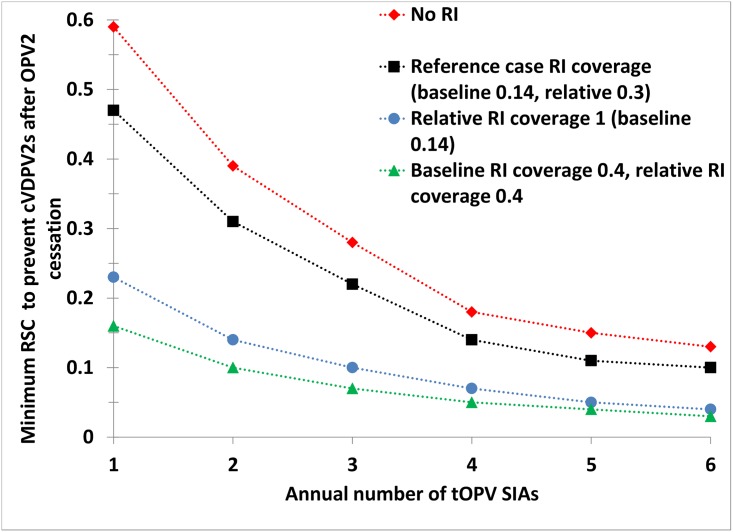
Interaction between tOPV SIA quality, SIA frequency, and RI coverage in northwest Nigeria. Acronyms: cVDPV, circulating vaccine-derived poliovirus; OPV2, serotype 2-containing oral poliovirus vaccine; RI, routine immunization; RSC, relative SIA coverage in the under-vaccinated subpopulation; SIA, supplemental immunization activity; tOPV, trivalent oral poliovirus vaccine.

Fig 3 considers the impacts of varying levels of RI coverage, while still maintaining SIA coverage at 85%. Not surprisingly, without any RI, preventing cVDPV2s after OPV2 cessation requires more tOPV SIAs, higher RSC, or both compared to the reference case. If RI coverage in the subpopulation increases to the level of the general population (i.e., relative RI coverage of 1), then this results in a notable downward curve shift, implying lower demands on RSC and/or SIA frequency to prevent cVDPV2s after OPV2 cessation. With a moderate improvement in the baseline RI coverage to 0.4 and with a relative RI coverage of 0.4, 2 annual tOPV SIAs remain sufficient to prevent cVDPV2s after OPV2 cessation even if RSC decreases to 0.1. If RI coverage improves to 0.5 in the general population and RSC to 0.5 (i.e., resulting in an absolute coverage of 0.25 in the under-vaccinated subpopulation), then this significantly slows down the accumulation of new susceptible children and loss of overall population immunity. Consequently, for this level of RI coverage, a single annual SIA with high quality in the general population can prevent cVDPV2s after OPV2 cessation, even if it does not reach the subpopulation. However, this finding depends on high SIA coverage in the general population and on residual immunity derived from the outbreak and response that occurred in 2014; without these conditions, preventing cVDPV2s after OPV2 cessation may require higher RI coverage.

## Discussion

Achieving and maintaining high population immunity for all 3 serotypes remains critical for both sustained WPV elimination and successful management of OPV cessation.[10, 24, 31, 34] The safest and most efficient way to successfully eradicate WPV and transition to the post-OPV era involves more intense vaccination than the strict minimum needed to reach the threshold population immunity level.[24, 35] Investments made to improve RI and/or SIAs all affect population immunity and reduce the risk of cVDPVs after OPV cessation. While small numerical increments in RI or SIA quality can yield the same effect on population immunity and cVDPV risks as added SIA rounds, these may represent large increases in practice, which require active outreach activities and extensive efforts to identify under-served population previously missed by population censuses and vaccination campaigns,[36] sometimes in the context of security challenges. Program managers should compare the feasibility and cost of these different options, particularly related to reaching under-vaccinated subpopulations compared to and in addition to conducting frequent rounds. Programs should focus on overall performance and ensure that they achieve sustained population immunity well above the threshold everywhere and for each serotype through cessation of homotypic OPV.[37] Although our analysis of SIAs focused primarily on the importance of reaching the under-vaccinated subpopulations given the range of SIA coverage considered in the exploratory sensitivity analyses, maintaining high SIA coverage in the general population represents a critical factor that also affects the results and remains essential in efforts to sustain overall population immunity and prevent outbreaks of imported poliovirus or cVDPVs.[8]

Our finding of the limited impact of IPV on population immunity to transmission, and thus its ability to prevent cVDPV emergence remains consistent with our earlier findings [33] and the literature on IPV-induced immunity,[27, 28] including a recent study that showed that IPV provides a better boost in individual intestinal immunity than bOPV.[38] Our model assumes that both successful IPV immunization and successful OPV immunization “takes” return individuals with prior potentially waned live poliovirus-induced immunity to the highest state of immunity in the model.[25] However, for OPV, the model uses an effectively lower “take” of the booster dose given oral administration by accounting for the relative susceptibility of the prior immunity state (i.e., between 0.2 for recent immunity and 0.8 for the last waning stage), while for an IPV dose we assume no reduction in the “take” due to prior immunity.[25] In addition, we assume a higher per-dose take rate for IPV than bOPV (i.e., 0.63 vs. 0.54 in the Nigeria model).[23] The significant difference in the effective take of IPV compared to bOPV explains the significant difference in the intestinal immunity observed at the individual level in a recent clinical trial.[38]

At the population level, which represents the critical perspective for poliovirus transmission and overall national immunization program performance,[37] our model suggests only a moderate role of individuals with prior live poliovirus-induced immunity on overall population immunity, with fully susceptible individuals driving the overall population immunity to transmission, particularly since they mix preferentially within their age group and/or subpopulation.[10, 23] Numerous prior studies demonstrated very limited intestinal immunity provided by IPV-only in fully susceptible individuals,[27, 39, 40] which implies a very limited impact of IPV alone on population immunity in settings conducive to fecal-oral poliovirus transmission.[33, 41–43] Moreover, OPV provides additional population immunity to transmission through secondary immunization of vaccine recipients while IPV does not. Consequently, despite the better ability of IPV to boost individual intestinal immunity in individuals with prior live poliovirus-induced immunity, our analyses suggest little overall impact of low-coverage IPV RI on population immunity in northwest Nigeria. Further analyses of IPV coupled with higher RI coverage or used in SIAs remains beyond the scope of this work, but we anticipate only marginal improvements given the inherent properties of IPV. Nevertheless, after OPV cessation, IPV will represent the only available poliovirus vaccine to provide some measure of population immunity to transmission in the long-term and to prevent paralytic cases in the event of a poliovirus reintroduction.

Many model inputs remain uncertain. However, changing the model inputs not directly related to vaccination from 2015 forward might lead to results inconsistent with the past experience in northwest Nigeria and would require refitting the model. We focused on changes of the vaccination inputs with all other inputs held constant, and consequently we did not consider the uncertainty about the non-vaccination inputs in our analyses. Although the reference case estimates of the vaccination inputs remain uncertain, including the relative RI and SIA coverage in the under-vaccinated subpopulation, the process of fitting the northwest Nigeria model to the actual experience provides some confidence that the combination of model inputs gives an adequate approximation of the situation in this conceptual subpopulation.[23, 25] We recognize that translating the conceptual under-vaccinated subpopulation into real people in the field may prove difficult. For example, in reality the under-vaccinated subpopulation, which we estimated as 10% of the total Nigerian northwest population, may include unevenly distributed pockets of communities in hard-to-reach rural areas, as well as under-served urban groups, and as the coverage in these population improves the size of the truly under-vaccinated population decreases.

Despite the uncertainties, the model confirms the experience of other countries (most notably India) that did not succeed in eliminating WPV until they identified and vaccinated large clusters of under-vaccinated people that sustained WPV transmission.[44] Continuing to improve vaccination coverage among historically under-vaccinated groups in northwest Nigeria can greatly facilitate the transition to OPV2 cessation and decrease the overall burden of paralytic polio. Without these improvements, a high frequency of tOPV rounds until OPV2 cessation and bOPV rounds until OPV13 cessation remains necessary. Thus, the use of tOPV for SIAs continues to represent an important consideration. While Nigeria must maintain high population immunity for serotype 1 to stop WPV1 and prevent any subsequent WPV1 importations, doing so at the expense of immunity to serotype 2 may delay or undermine successful OPV2 cessation. While, using more tOPV at the expense of more bOPV must not undermine serotype 1 and 3 immunity, bOPV SIAs provide no boost in population immunity to transmission at all for serotype 2, whereas tOPV SIAs provide a considerable boost in population immunity to transmission for serotypes 1 and 3. Further modeling studies can help inform decisions related to choices of the number of bOPV vs. tOPV SIAs leading up to OPV2 cessation and bOPV SIAs quality and frequency leading up to OPV13 cessation,[45] and the optimal timing of SIAs relative to OPV cessation and seasonality. In addition, they might expand these analyses to consider other high-risk populations in the world (e.g., those with relatively higher R_0_ and lower OPV take rates).

## Conclusions

To achieve and maintain sufficiently high population immunity to transmission and prevent reintroduction of WPVs and cVDPVs in northwest Nigeria, national immunization program managers should consider the interactions of immunization activities as they allocate resources to strengthening RI services and increasing the frequency and quality of SIAs. Improving the quality of RI and SIAs to reach under-vaccinated groups reduces the frequency of SIAs needed to maintain high enough population immunity. While the model assumptions for northwest Nigeria, including the characteristics of the under-vaccinated subpopulation may differ for high-risk populations in different areas of the world, the qualitative insights of this work extend to all high-risk populations.
